# Diversity in Naturally Acquired Immunity to Group B Streptococcus: A Comparative Study of Women From Bangladesh, Malawi, and the United Kingdom

**DOI:** 10.1093/infdis/jiae607

**Published:** 2024-12-18

**Authors:** Shadia Khandaker, Shilpee Sharma, Tom Hall, Suzanna Lim, Janne Lehtonen, Stephanie Leung, Zabed Bin Ahmed, Andrew Gorringe, Samir K Saha, Arnaud Marchant, Kirsty Le Doare, Aras Kadioglu, Neil French

**Affiliations:** Department of Clinical Infection, Microbiology and Immunology, University of Liverpool, Liverpool, United Kingdom; European Plotkin Institute for Vaccinology, ULB Centre for Research in Immunology, Université libre de Bruxelles, Brussels, Belgium; Centre for Neonatal and Paediatric Infection, Institute of Infection and Immunity, St George's, University of London, London, United Kingdom; Centre for Neonatal and Paediatric Infection, Institute of Infection and Immunity, St George's, University of London, London, United Kingdom; MinervaX ApS, Copenhagen, Denmark; United Kingdom Health Security Agency, Salisbury, United Kingdom; Child Health Research Foundation, Dhaka, Bangladesh; United Kingdom Health Security Agency, Salisbury, United Kingdom; Child Health Research Foundation, Dhaka, Bangladesh; European Plotkin Institute for Vaccinology, ULB Centre for Research in Immunology, Université libre de Bruxelles, Brussels, Belgium; Centre for Neonatal and Paediatric Infection, Institute of Infection and Immunity, St George's, University of London, London, United Kingdom; United Kingdom Health Security Agency, Salisbury, United Kingdom; Makerere University–Johns Hopkins University, Kampala, Uganda; World Health Organization, Geneva, Switzerland; Department of Clinical Infection, Microbiology and Immunology, University of Liverpool, Liverpool, United Kingdom; Department of Clinical Infection, Microbiology and Immunology, University of Liverpool, Liverpool, United Kingdom; Malawi Liverpool Wellcome Clinical Research Programme, Blantyre, Malawi

**Keywords:** Alp protein, antibody diversity, capsular polysaccharide, group B *Streptococcus*, mouse model

## Abstract

**Background:**

Significant disparities in group B *Streptococcus* (GBS) colonization and neonatal disease rates have been documented across different geographic regions. For example, Bangladesh reports notably lower rates as compared with the United Kingdom and Malawi. This study investigates whether this epidemiologic variability correlates with the immune response to GBS in these regions.

**Methods:**

Qualitative and quantitative analyses of naturally acquired immunoglobulin G (IgG) antibodies against GBS capsular polysaccharide and the Alp protein family were conducted in serum samples from women of childbearing age in the United Kingdom, Bangladesh, and Malawi. The efficacy of these antibodies in clearing vaginal colonization or protecting newborns from GBS infection was assessed with humanized mouse models.

**Results:**

Bangladeshi women displayed the highest diversity in serotype distribution, with elevated IgG levels in the serum against GBS capsular polysaccharides Ia, Ib, II, III, IV, and V, as well as Alp family proteins. In contrast, Malawian sera demonstrated the weakest antibody response. Bangladeshi sera also showed heightened IgG-mediated complement deposition, opsonophagocytic killing, and neonatal Fc receptor binding while tested against capsular polysaccharide Ib. In a humanized neonatal Fc receptor mouse model, Bangladeshi sera led to faster clearance of GBS virulent serotype Ib vaginal colonization. Additionally, offspring from dams passively immunized with Bangladeshi sera demonstrated notably increased survival rates.

**Conclusions:**

This study demonstrates significant variability in the immune response to GBS across different geographic regions. These findings underscore the importance of understanding GBS-induced immune response in diverse populations, which may significantly affect vaccine efficacy in these regions.

## Teaser

This study highlights distinct immune responses to group B *Streptococcus* in women across the United Kingdom, Bangladesh, and Malawi, revealing regional differences that may affect colonization clearance and neonatal protection, with implications for vaccine effectiveness across diverse populations.

Group B *Streptococcus* (GBS) is a major cause of neonatal and infant mortality worldwide [1]. Although initiatives are underway to develop effective GBS vaccines, baseline immunity against GBS in the population is largely unknown. Several studies have reported a striking discrepancy in GBS colonization and disease incidences across geographic regions, with variability observed in maternal rectovaginal colonization rates and neonatal disease incidences across Europe, Africa, and Southeast Asia [2–4]. For instance, the maternal rectovaginal colonization rate was reported as 21.2% in Malawi and 20% in the United Kingdom, whereas in Bangladesh it was 11%, though more recently reported as 17.5% by Kwatra et al [4–7]. Infant GBS disease incidence is significantly higher in the United Kingdom and Malawi than in Bangladesh: 0.94 per 1000 live births in the United Kingdom, 1.8 in Malawi, and only 0.10 in Bangladesh [2, 8, 9]. These intriguing differences highlight the necessity to explore the underlying factors contributing to variation in GBS epidemiology. Contributing factors include GBS strain virulence, genetic susceptibility, and environmental exposure; health care practices, such as antibiotic use and access; and regional differences in population immunity, whether from natural exposure or passively acquired anti-GBS antibodies, which may significantly shape the epidemiologic landscape [10, 11].

Increased levels of naturally occurring antibodies against GBS capsular polysaccharide (CPS) are associated with a lower risk of subsequent acquisition of rectovaginal colonization in pregnant women [12–14]. This association extends to a reduced risk of GBS early- and late-onset disease in newborns, underscoring the protective role of placentally transferred maternal antibodies that may remain effective up to 90 days after birth [15–17]. Placentally transferred, naturally occurring GBS protein–specific antibodies have also been linked to protection against early- and late-onset disease [18, 19]. In addition to antibody concentration, functional properties and subclass specificity of immunoglobulin G (IgG) are important determinants of protection against various pathogens and vaccines, including GBS [20–24]. Furthermore, the binding of IgG to neonatal Fc receptor (FcRn) influences the clearance of GBS vaginal colonization and transplacental transfer of antibodies [25, 26].

Of the 10 GBS serotypes, Ia, Ib, II, III, IV, and V are the most widespread worldwide and account for most colonization and invasive diseases [27]. Hence, these 6 CPS antigens have been included in a hexavalent GBS conjugate vaccine currently in phase 2 clinical trials [12, 28]. Alongside GBS polysaccharide–based vaccines, a protein subunit vaccine targeting the N-terminus of the GBS alpha-like protein (Alp) family of surface proteins—Alp1, Alp2/3, AlphaC (αC), and Rib—has been shown to elicit a functional antibody response in healthy adult nonpregnant women [23].

In the present study, we investigated naturally acquired anti-GBS antibodies in women of childbearing age, targeting CPS Ia, Ib, II, III, IV, and V, as well as the Alp-N, across 3 geographically distinct populations: the United Kingdom, Malawi, and Bangladesh. Through a quantitative and qualitative assessment of IgG using in vitro methods and clinically relevant in vivo models, we examined the correlation between the humoral immune response and the reported variations in GBS epidemiology across these regions.

## METHODS

### Study Design and Participants

Serum samples used in this study were collected from completed studies or a repository of publicly available samples stored anonymously with generic identification numbers at the University of Liverpool, United Kingdom. One hundred samples were randomly selected from each study site: the United Kingdom, Bangladesh, and Malawi. Inclusion criteria were women aged 25 to 35 years, regardless of GBS colonization or pregnancy status. All samples were collected or stored with informed consent from participants. Details of ethics are included in the supplementary materials.

### Quantitation of CPS-Specific IgG in Sera

A multiplex direct Luminex-based immunoassay was utilized to measure the levels of IgG antibodies in serum samples, as described elsewhere [29]. A brief description is given in the supplementary materials. Twelve Malawian samples were excluded due to insufficient quantity for this assay.

### Quantitation of Alp-N–Specific IgG in Sera

IgG against Alp1-N, Alp2/3-N, αC-N, and Rib-N antigens in the serum samples were quantified by enzyme-linked immunosorbent assay, as described previously [30]. See the supplementary file for a detailed method.

### Quantitation of CPS- and Alp-N–Specific IgG Subclasses

Fluorescent magnetic beads (Luminex Corp) were coupled with CPS Ib and Alp-N antigens following the manufacturer's instruction. Antigen-coated beads (5 × 10^2–3^ beads/well, 80 μL) were mixed with 20-μL serum samples prediluted in assay wash buffer (phosphate-buffered saline [PBS] 1×, 0.1% bovine serum albumin [BSA], 0.05% Tween 20, pH 7.4) in a 96-well plate and incubated for 2 hours at room temperature on an orbital shaker (450 rpm). Sample dilutions are 1:250 for IgG1, IgG2, and IgG3 and 1:50 for IgG4. R-Phycoerythrin (RPE)-coupled detection antibodies for each subclass (Southern Biotech) were added at a concentration of 0.65 μg/mL and incubated for 1 hour at room temperature on an orbital shaker. Beads were then washed and thoroughly mixed in the buffer by briefly vortexing and sonicating for 30 to 60 seconds. The binding between antigens and antibodies was quantified by the BioPlex-200 system (Bio-Rad), with results reported as mean fluorescence intensity (MFI).

### Antibody-Dependent Complement Deposition Assay

Total IgG in serum samples was purified with the Melon Gel IgG Purification Kit (Thermo Fisher Scientific) following the manufacturer's instructions. CPS Ib and Alp protein antigen-coated beads (5 × 10^2–3^ beads/well, 5 μL) were incubated with 80 μL of purified IgG at a final dilution of 1:250, followed by a 2-hour incubation at 37 °C. After washing 4 times with wash buffer (PBS + 1% BSA), a 1:150 dilution of human complement (S1764; Sigma) was added and further incubated for 30 minutes at 37 °C. Subsequently, biotinylated monoclonal anti-human C3d (1 µg/mL in PBS, 80 μL; A702 [Quidel]) was added and incubated for 30 minutes at room temperature in the dark. After washing, streptavidin-RPE (1 µg/mL in PBS) was added, followed by another 30 minutes of incubation at room temperature in the dark. Finally, the beads were washed and resuspended in wash buffer, and C3d binding was measured by the BioPlex-200 system, with results expressed as MFIs.

### FcRn-Binding Assay

Recombinant Human FCRN Protein (8639-FC; R&D) was biotinylated with the EZ-Link Sulfo-sNHS-LC-Biotin labeling kit (Thermo Fisher). Streptavidin-Phycoerythrin (PE) (PJ31S; Prozyme) was added to FcRn-biotin (0.65 µg/mL) at 4:1 to create a PE-conjugated FcRn detection reagent. After the addition of free biotin (1% v:v), the reagent was stored at 4 °C in the dark until use.

CPS Ib or Alp-N antigen-coated beads (5 × 10^2–3^ beads/well, 80 μL) were mixed with 20 μL of serum in a black 96-well microplate and incubated for 2 hours at room temperature on an orbital shaker (450 rpm) in the dark. Following incubation, beads were washed with the assay buffer (PBS, 0.1% BSA, 0.05% Tween-20, pH 7.4), and the previously prepared detection reagent was added and further incubated for 1 hour. After the final wash in the wash buffer adjusted to pH 5.8, beads were resuspended in the same buffer to measure the FcRn binding with IgG, which was measured with the BioPlex-200 system, and the results were expressed in MFIs.

### Opsonophagocytosis Killing Assay

The opsonophagocytosis killing (OPK) assay was performed via the HL-60 cell line as described by Leung et al [31]. See the supplementary file for a detailed method.

### Mouse Model of Passive Immunization, Vaginal Colonization, and Neonatal Protection

Serum samples from the United Kingdom, Malawi, and Bangladesh were pooled within each country group and normalized with PBS to achieve a consistent IgG concentration of 6 µg/mL. Details on the selected serum samples are provided in Supplementary Table 1. All animal experiments were performed per regulations of the Home Office Scientific Procedures Act (1986; project licence PP2832279) and the University of Liverpool Ethical and Animal Welfare Committee.

Female BALB/c mice aged 6 to 8 weeks were intraperitoneally injected with 0.5 mg of β-oestradiol suspended in 100 µL of sesame oil. The following day, mice received intraperitoneal injections of 100-µL serum samples or PBS. Twelve hours later, mice were vaginally inoculated with 1 × 10^5^ colony-forming units (CFU) of GBS serotype Ib, a clinical strain isolated from a neonatal meningitis case and tested for virulence in a murine model. Vaginal tissue was collected from mice euthanized on days 1, 3, and 7 postinoculations and then homogenized, serially diluted in PBS, and plated on blood agar. CFU values per milliliter were enumerated after overnight incubation at 37 °C to determine bacterial load.

Humanized FcRn mice (014565B6.Cg-FcgrttmlDcr Tg[FCGRT]32Dcr/DcrJ), purchased from the Jackson Laboratory, were bred in-house for the neonatal protection model. Pregnant dams (n = 5/country) were injected intraperitoneally with serum samples or PBS on day 16 of gestation. Pups were challenged intraperitoneally with GBS within 24 hours of birth and monitored for disease signs and mortality over 72 hours. To calculate the IgG transfer ratio, a blood sample was collected from another batch of humanized FcRn pregnant mice after 24 hours of passive immunization via tail bleeding and from the pups within 12 to 18 hours of birth via the chest cavity after dissection of the heart. IgG concentration to CPS Ib was measured with the immunoassay described previously.

### Statistical Analysis

Statistical analyses were conducted with Prism version 9.0.1 (GraphPad). Differences across 3 countries were determined by the nonparametric Kruskal-Wallis 1-way analysis of variance test with Dunn multiple-comparison test. Two-way analysis of variance with Dunnett post hoc test was used to compare CFU between the 3 countries and PBS control. Survival curves were compared by the log-rank test (Mantel-Cox). For all analyses, an adjusted *P* < .05 was considered significant to account for multiple comparisons and control for type I errors. Reverse cumulative distribution curves were created by Microsoft Excel version 1808 to present the IgG concentrations against CPS and Alp antigens across countries.

## RESULTS

### Serotype Diversity and Quantity of GBS CPS-Specific Antibody

**Figure 1.**
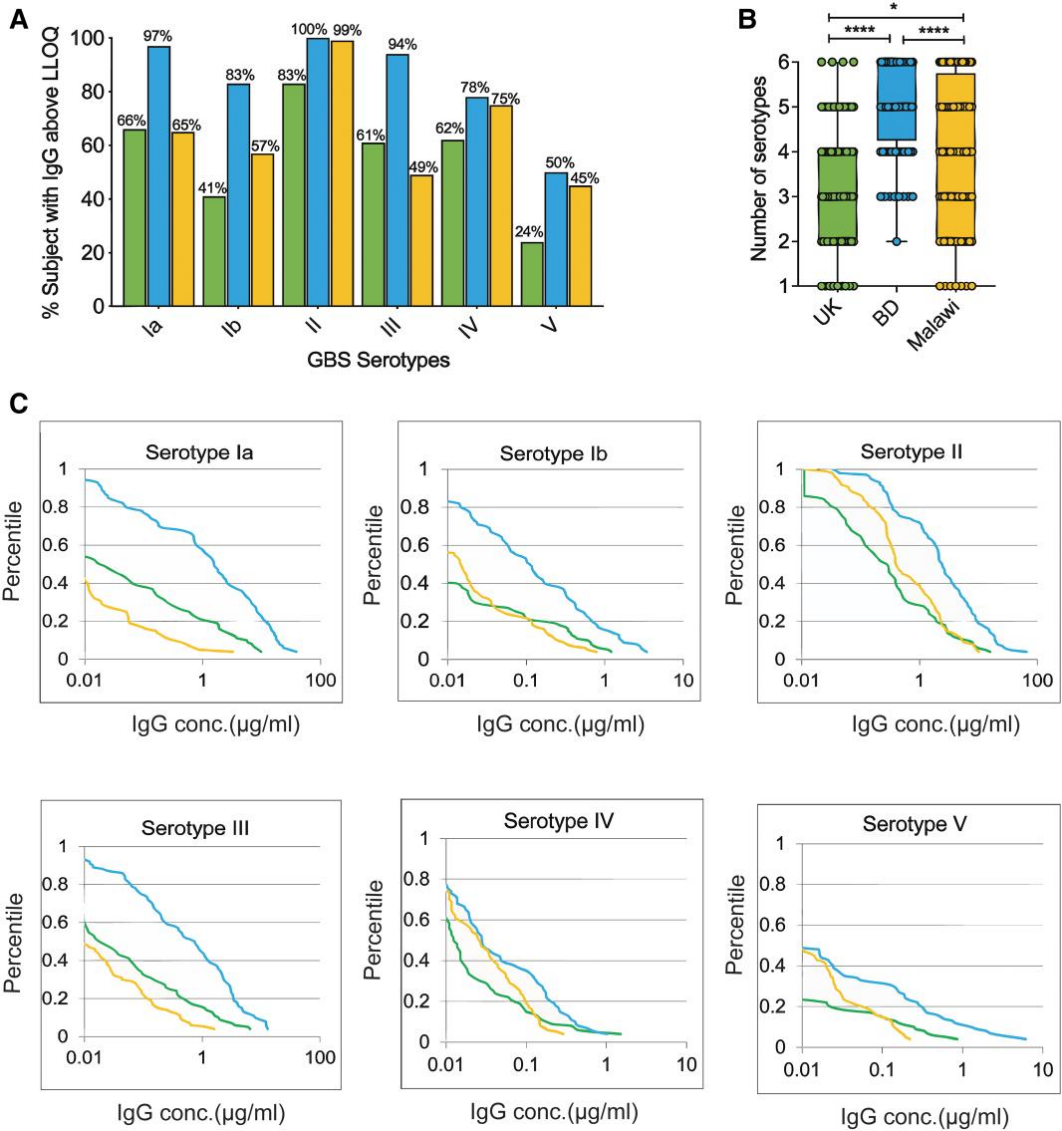
Diverse seroprevalence to group B *Streptococcus* (GBS) capsular polysaccharide observed in the United Kingdom, Bangladesh, and Malawi. *A*, Presence of immunoglobulin G (IgG) against GBS serotypes Ia, Ib, II, III, IV, and V in serum samples collected from the United Kingdom (n = 100), Bangladesh (n = 100), and Malawi (n = 88). The bar plot represents the percentage of women with IgG concentration equal to or above the lower limit of quantification (LLOQ) for the assay. *B*, Simultaneous presence of IgG antibodies against multiple serotypes of GBS in a single woman in 3 countries. The analysis included serum samples with a concentration equal to or above the LLOQ. Box and whisker plots indicate median, IQR, and minimum/maximum values. *P < .05. ****P < .0001. *C*, Reverse cumulative distribution curves show IgG levels in the serum samples. Analysis included all serum samples in the study. Sample concentrations below the LLOQ were assigned half the LLOQ value for the corresponding serotypes. Green, blue, and yellow lines represent the sera from the United Kingdom, Bangladesh, and Malawi, respectively. BD, Bangladesh.

We conducted a comprehensive analysis of naturally developed antibodies against GBS in women aged 25 to 35 years residing in 3 geographically diverse regions: the United Kingdom (n = 100), Bangladesh (n = 100), and Malawi (n = 88). Serum samples were evaluated for IgG concentration against GBS CPS Ia, Ib, II, III, IV, and V. Bangladeshi women exhibited the highest seroprevalence against all 6 serotypes, with serotype II–specific antibodies being the most abundant across all 3 countries (Figure 1*A*). Multiple GBS serotype–specific antibodies were detected in women from all regions, with a notably higher number in Bangladeshi women (Figure 1*B*). Approximately 31% of Bangladeshi women had IgG against all 6 serotypes, as compared with 23% in Malawi and only 4% in the United Kingdom. Reverse cumulative distribution curves revealed higher antibody concentrations in Bangladeshi serum for all 6 serotypes (Figure 1*C*). The geometric mean concentration of IgG against the 6 serotypes was highest in Bangladeshi sera (Supplementary Table 2). Overall, Bangladeshi women demonstrated the highest seroprevalence, presence of IgG against multiple CPS types, and IgG concentrations against all 6 serotypes assessed in this study.

### Distribution of the CPS Ib–Specific IgG Subclasses and Effector Functions Across 3 Countries

**Figure 2.**
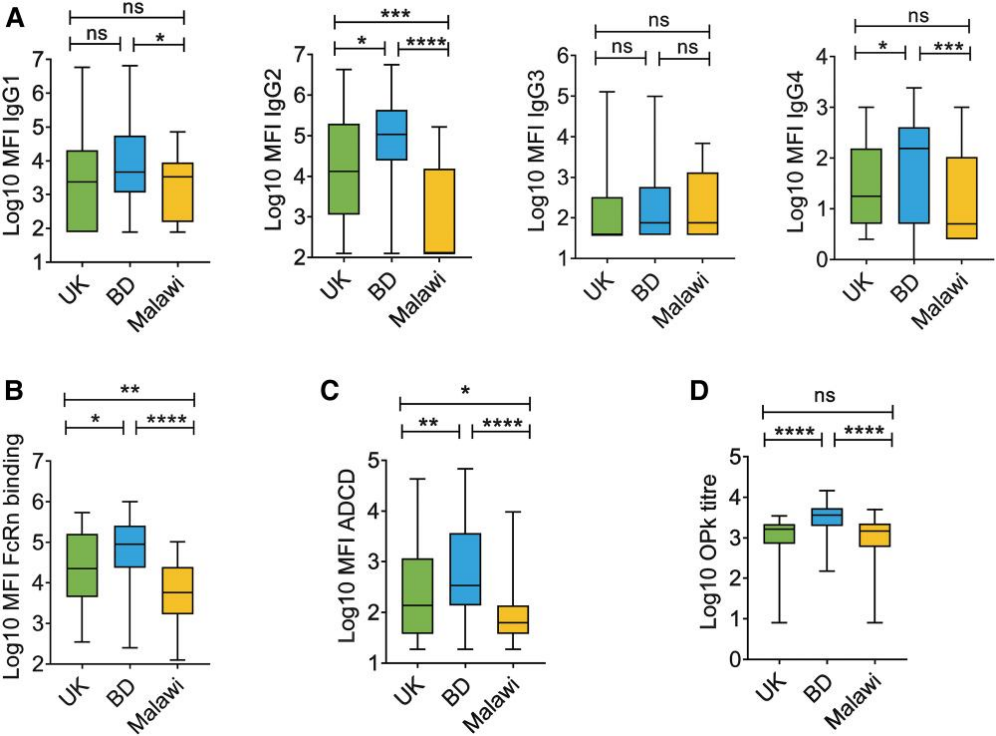
Subclass distribution of capsular polysaccharide (CPS) Ib–specific immunoglobulin G (IgG) and IgG functionality across 3 countries shows dominance of the Bangladeshi sera. *A*, Group B *Streptococcus* CPS Ib–specific IgG subclasses (IgG1, IgG2, IgG3, and IgG4) in serum samples collected from the United Kingdom, Bangladesh, and Malawi (n = 55/country). Data are depicted as mean fluorescence intensity (MFI). *B*–*D*, Effector functions of CPS Ib–specific IgG were determined as neonatal Fc receptor (FcRn) binding, antibody-dependent complement deposition (ADCD), and opsonophagocytic killing (OPK) in the serum samples (n = 55/country). The Luminex-based assay was used to measure FcRn binding and ADCD, and the results are expressed as MFI. OPK was determined by in vitro OPK assay, and the results are expressed as OPK titer, determined as 50% killing by HL60 neutrophils vs no-serum control. Samples with titers below the lowest value were assigned half the minimum detectable titer. Statistical analysis to evaluate intercountry variation was performed by a nonparametric Kruskal-Wallis test with Dunn multiple-comparison tests. *P < .05. **P < .01. ***P < .001. ****P < .0001. Box and whisker plots indicate median, IQR, and minimum/maximum values. BD, Bangladesh; ns, not significant.

Upon analysis of IgG subclass prevalence in the serum samples, the MFI of IgG1 was lower in Malawi sera as compared with the United Kingdom (not statistically significant) and Bangladesh (*P* < .05). The most significant difference was found in IgG2 levels, with the Malawian population exhibiting substantially lower IgG2 values than the United Kingdom and Bangladesh (Figure 2*A*). Bangladeshi sera displayed significantly higher IgG4 levels, while IgG3 levels remained similar across all 3 countries.

Bangladeshi women exhibited notably higher levels of FcRn binding and antibody-dependent complement deposition (ADCD), whereas Malawi sera demonstrated the lowest ADCD and FcRn-binding capabilities (Figure 2*B*  and 2*C*). Additionally, Bangladeshi sera displayed the highest OPK response against the CPS Ib–expressing GBS strain (14092; NCTC), while comparable titers were observed in UK and Malawi sera (Figure 2*D*). These results indicate enhanced IgG-mediated effector functions and FcRn binding in Bangladeshi women in response to GBS.

### Seroprevalence, Subclass Distribution, and Effector Functions of Alp-N–Specific IgG Across 3 Countries

**Figure 3.**
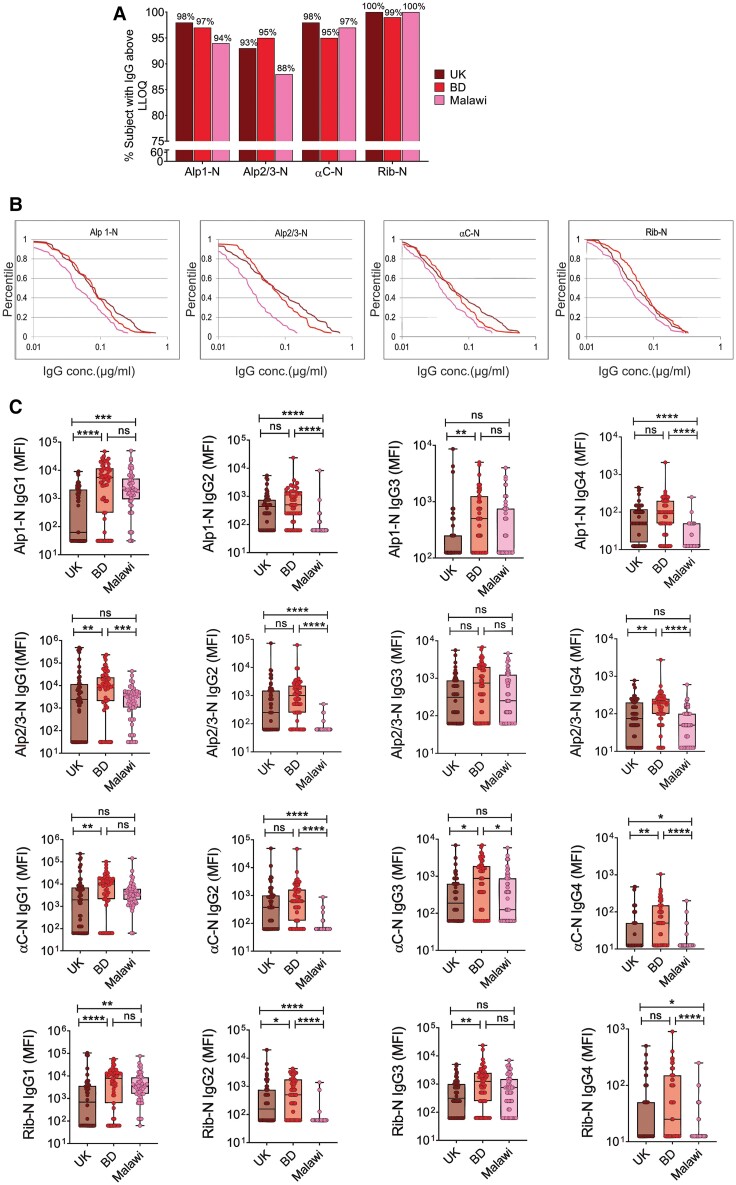
Differential seroprevalence and immunoglobulin G (IgG) subclass distribution observed against Alp-N–specific IgG across the countries. *A*, IgG against group B *Streptococcus* (GBS) Alp proteins in the serum samples from the United Kingdom, Bangladesh, and Malawi (n = 100/country) where the bar plot represents the percentage of women possessing IgG above or equal to the lower limit of quantification (LLOQ) against GBS surface protein antigens Alp1-N, Alp2/3-N, αC-N, and Rib-N. *B*, Reverse cumulative distribution curves show IgG levels against GBS Alp proteins in the serum samples of all study participants (n = 100/country). Sample concentrations below the LLOQ were assigned half the LLOQ value of the corresponding serotypes. Maroon, red, and pink lines represent the sera from the United Kingdom, Bangladesh, and Malawi, respectively. *C*, Subclass distribution of Alp-specific IgG in the serum samples (n = 55/country) where data are presented as mean fluorescence intensity (MFI). Statistical significance was determined by the Kruskal-Wallis test with Dunn multiple-comparison test. *P < .05. **P < .01. ***P < .001. ****P < .0001. Box and whisker plots indicate median, IQR, and minimum/maximum values. Alp, alpha-like protein; BD, Bangladesh; ns, not significant.

Subsequently, we profiled the naturally acquired humoral response against the GBS surface proteins of the Alp family (n = 100/country). No significant variations were noted in the prevalence of anti-Alp-N antibodies among the 3 countries, with high Rib-N seropositivity observed across all countries. However, a notably lower seropositivity against Alp2/3-N was evident in the Malawian population (Figure 3*A*). The reverse cumulative distribution curves for antibody concentrations in Figure 3*B* depict a significantly lower Alp-N–specific IgG concentration in Malawi as compared with the United Kingdom and Bangladesh. Significant differences were observed in geometric mean concentrations across the 3 countries, with the Malawian population displaying the lowest antibody levels (Supplementary Table 3).

Although the IgG subclasses were comparable for some Alp proteins among the 3 countries, a general trend of higher subclass levels was observed in Bangladeshi sera, particularly in IgG1 and IgG3 (Figure 3*C*). In contrast, Malawi exhibited the lowest levels for all subclasses against Alp-N proteins, with a significantly low IgG2 level as compared with the United Kingdom and Bangladesh (Figure 3*C*).

**Figure 4.**
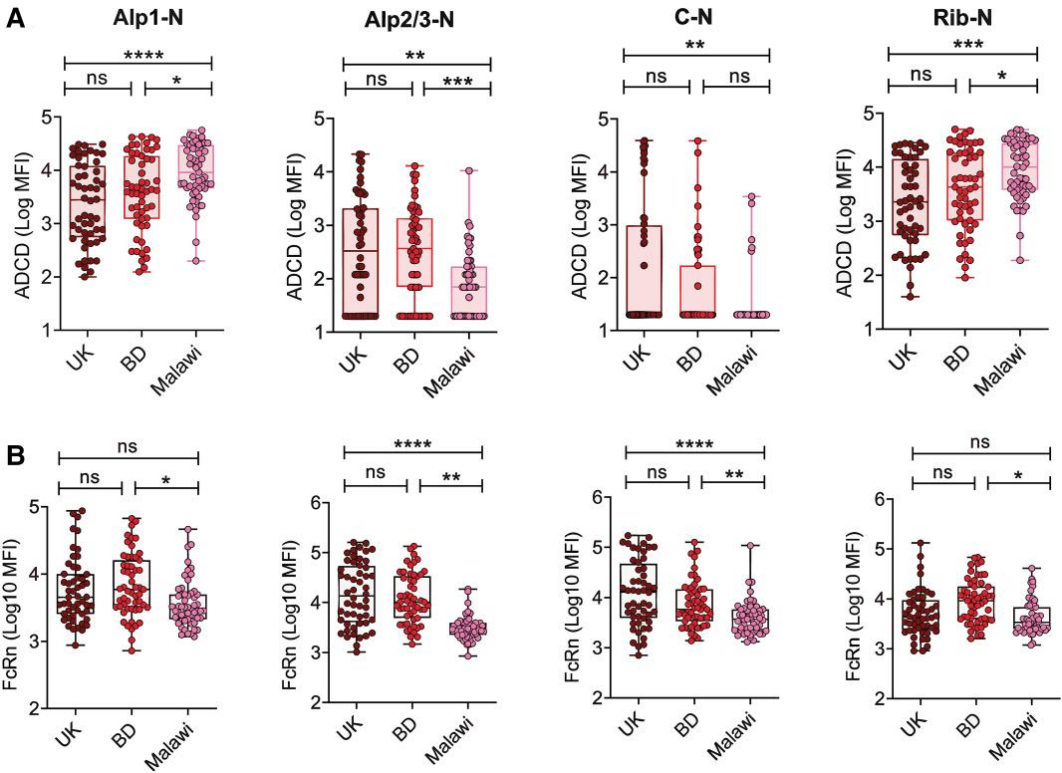
Variable immunoglobulin G (IgG) effector functions observed against Alp-N proteins in different country settings. *A*, Antibody-dependent complement deposition (ADCD) exerted by IgG against Alp1-N, Alp2/3-N, αC-N, and Rib-N proteins in the serum of women from the United Kingdom, Bangladesh, and Malawi. *B*, Neonatal Fc receptor (FcRn) binding of IgG against 4 Alp proteins across the 3 countries. Statistical significance was determined by Kruskal-Wallis test with Dunn multiple-comparison test. *P < .05. **P < .01. ***P < .001. ****P < .0001. n = 55/country for both assays. Box and whisker plots indicate median, IQR, and minimum/maximum values. Alp, alpha-like protein; BD, Bangladesh; MFI, lower limit of quantification; ns, not significant.

Among the 4 Alp-N proteins, the greatest ADCD was exhibited by IgG specific for Alp1-N and Rib-N (Figure 4*A*). Interestingly, ADCD to Alp1-N and Rib-N antigens was significantly higher in Malawi than in other countries. In contrast to ADCD, FcRn binding by anti-Alp protein IgG was similar among all 4 protein antigens, although there were marked intercountry variations (Figure 4*B*). Across all antigens, the lowest FcRn binding was observed in Malawi, most notably with Alp2/3-N– and αC-N–specific IgG, with no significant difference between the United Kingdom and Bangladesh.

Overall, Malawian sera had the lowest levels of Alp-N–specific IgG, while Bangladeshi sera showed a stronger IgG1 and IgG3 response when compared with other countries. Variable antibody effector functions were observed across the 3 countries for different Alp proteins.

### Effect of Passive Immunization With Sera in Mouse Vaginal GBS Colonization and Neonatal Protection

**Figure 5.**
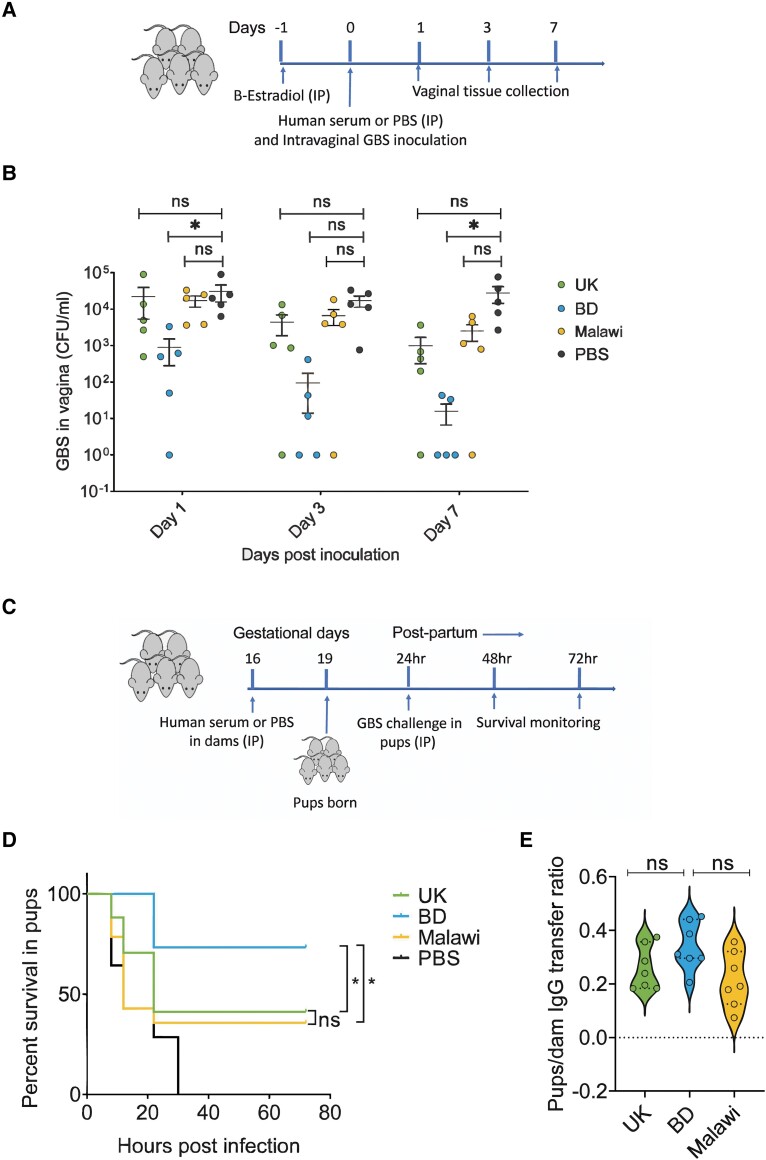
Bangladeshi serum conferred superior protection against group B *Streptococcus* (GBS) vaginal colonization and neonatal sepsis. *A*, Schematic representation of GBS vaginal colonization in female BALB/c mice treated with serum samples (n = 5/group). *B*, Bacterial load in the mouse vaginal tissues is plotted, where each dot represents a single mouse. Data are expressed as mean ± SEM. Colony-forming units (CFU) in the serum-treated groups were compared with the group treated with phosphate-buffered saline at each time point by 2-way analysis of variance with Dunnett test for multiple comparisons. *P < .05. *C*, Schematic representation of neonatal protection from GBS infection following passive immunization of pregnant mice with serum samples (n = 5/group). *D*, Kaplan-Meier survival curve shows the survival of pups born from dam that received the UK sera (n = 17), Bangladeshi sera (n = 15), and Malawi sera (n = 14) or PBS (n = 14). The survival curves were compared by the log-rank Mantel-Cox test. Data were pooled from 3 independent experiments. *E*, The violin plot shows the IgG transfer ratios of pups to dam for the United Kingdom, Bangladesh, and Malawi serum measured in humanized neonatal Fc receptor pregnant mice (n = 3 per country) and their pups (n = 7 per country). Statistical analysis was performed by Kruskal-Wallis 1-way analysis of variance with Dunn test for multiple comparisons. BD, Bangladesh; IP, intraperitoneal; ns, not significant.

Following significant variation observed in CPS- and Alp-specific functional antibodies across 3 countries, we evaluated the serum samples in protection against GBS colonization in a mouse model of vaginal colonization. Bangladeshi sera initiated bacterial clearance within 24 hours postinoculation, contrasting with no clearance observed in mice treated with the UK or Malawian sera or in the PBS control group (Figure 5*B*). By day 7, only 2 mice in the Bangladeshi sera group had a low GBS count, while PBS-treated mice retained a high bacterial load. Although colonization density was reduced in mice treated with the UK and Malawi sera, 80% remained colonized with GBS at day 7.

Subsequently, we assessed protective efficacy in a humanized FcRn mouse model. Pregnant mice were passively immunized with sera from the 3 countries, and pups were challenged with a lethal dose of serotype Ib GBS postbirth. Bangladeshi sera provided significantly higher protection than sera from the United Kingdom (*P* = .03) and Malawi (*P* = .01), with >70% pup survival as opposed to approximately 40% with UK and Malawian sera (Figure 5*D*). Although not statistically significant, Bangladeshi sera demonstrated a higher transfer ratio of IgG against CPS Ib to the pups (Figure 5*E*).

In summary, these results underscore the superior efficacy of Bangladeshi sera in reducing vaginal GBS colonization and protecting mouse pups from lethal GBS infection.

## DISCUSSION

In this study, we identified significant diversity in the seroprevalence of antibodies specific for GBS CPSs and surface Alp proteins among 3 geographically distinct areas: the United Kingdom, Bangladesh, and Malawi. We found notable variations in CPS- and Alp-specific antibody concentrations and their functional properties, with Bangladeshi women showing the highest response and Malawian women the lowest, particularly in CPS-specific antibodies. Moreover, Bangladeshi sera exhibited a more rapid reduction in GBS vaginal colonization and provided the most significant protection against GBS infection in neonates in a humanized mouse model. Our in vitro and in vivo findings may not apply to all GBS serotypes, as these assays were conducted with only serotype Ib, 1 of the 6 most prevalent serotypes in maternal colonization and disease in infants [4, 7, 32–34].

Our study demonstrates distinctive serotype-specific antibody responses across the United Kingdom, Bangladesh, and Malawi, shedding light on variations in GBS immune profiles in these regions. Our data suggest that a multivalent GBS vaccine might elicit strong responses to serotype II while generating a relatively lower response against serotype V across all 3 regions (Figure 1*A*). While we found high seroprevalence of the 2 predominant serotypes for maternal colonization, Ia and II, only serotype Ia is consistently reported as a significant cause of neonatal disease in these countries, and serotype II is less frequently associated with disease [5–9, 32, 35]. Additionally, Bangladeshi women exhibited significantly higher antibody titers against serotype III despite its low detection in Bangladesh. These variations between serotype prevalence in carriage or disease and immune responses may reflect the influence of factors beyond capsular types, such as specific sequence types and clonal complexes, and the dynamic relationship between GBS exposure and resulting antibody profiles [14, 36–38].

Several studies have demonstrated a correlation between functional antibody concentration and GBS colonization in women. Fabbrini et al and Haeusler et al reported higher anti-GBS antibody levels in colonized women vs noncolonized women [37, 38]. Conversely, Kwatra et al showed that women with higher serotype-specific antibodies had a significantly lower likelihood of acquiring a new homotypic serotype, with high OPK titers associated with protection from colonization throughout the study [14]. A longitudinal study by Le Doare et al in Gambia found that mothers with increased C3b/iC3b deposition were less susceptible to GBS colonization [20]. The relationship between pathogen exposure and the antibody response is challenging to discern in our cross-sectional study. Consequently, whether the high antibody levels observed in the Bangladeshi population result from recent GBS colonization or prior repeated exposures remains unclear. The high prevalence of multiple CPS-specific antibodies in Bangladesh may suggest increased exposure, potentially leading to a durable antibody response and contributing to the lower colonization rates observed in this population (Figure 1*B*).

Significant intercountry variations in IgG levels were observed against GBS Alp proteins, but the differences were less pronounced than those of the anti-CPS antibodies (Figure 3*B*). This outcome was anticipated since >99% of GBS isolates possess at least 1 of the Alp proteins, leading to the production of Alp-reactive antibodies regardless of CPS types [39, 40]. Notably, the heightened antibody response observed in Bangladesh and the diminished response in Malawi appear to be specific to GBS, as significantly elevated IgG levels were observed in Malawian women to other antigens, such as tetanus toxoid and cytomegalovirus glycoprotein B (Supplementary Figure 1).

Malawian women exhibited significantly reduced antibody-mediated effector functions against CPS Ib and most Alp proteins (Figures 2*B*–*D* and 4), which may correlate with lower levels of IgG1 and IgG2 against the CPSs and IgG1 and IgG3 against Alp proteins (Figures 2*A* and 3*C*). These IgG subclasses are vital for complement deposition and phagocytosis during infections with encapsulated bacteria such as pneumococci and GBS [24, 41, 42]. Notably, serum IgG levels may have influenced the variability in subclass composition and effector functions across countries. However, subclasses against CPS Ib and protein antigens did not consistently correlate with corresponding IgG concentrations (Supplementary Figures 2, 4–6). While CPS-specific ADCD and FcRn binding showed weak correlations with IgG concentrations, OPK did not, likely due to the presence of IgM, antibody avidity, or other serum inhibitory factors (Supplementary Figure 3) [21, 43–46]. The correlations between protein-specific IgG ADCD and FcRn binding were also variable (Supplementary Figures 7 and 8), potentially reflecting differences in Alp protein expression among prevalent GBS serotypes or other IgG structural properties.

The diversity observed in IgG quantity and functionality across the 3 countries was strongly reflected in GBS colonization and passive immunization outcomes in humanized FcRn mice. Bangladeshi sera demonstrated a substantial reduction in vaginal carriage density of GBS and provided enhanced protection against GBS invasive disease in mice (Figure 5). In addition to complement-mediated opsonophagocytic killing, the high FcRn-binding ability of IgG in Bangladeshi sera may have contributed to improved protection against vaginal colonization and invasive disease in mice. FcRn-mediated recycling and transcytosis of IgG not only prolong IgG half-life but have also been linked to GBS clearance from the mouse vaginal tract and efficient transplacental transfer of IgG in humans [25, 26]. However, since a significant amount of maternally derived IgG is transferred via breast milk, the protective effects observed in the pups may not be solely due to placental IgG transfer [47].

A significant limitation of our study is the lack of essential demographic information, such as details on women's GBS colonization or pregnancy status, recent antibiotic usage, and other comorbidities that could affect antibody responses to GBS [14, 48]. Additionally, the ethnic representation of the women in our UK cohort could not be determined retrospectively. However, we anticipate that the cohort mainly consists of White British individuals, as the majority of the population (90%) in Exeter, where the samples originated, identified as White, according to the 2021 census [49]. Future research incorporating detailed demographic and health data, including the GBS colonization status of the women, could help refine these findings further.

In conclusion, our study underscores a potential correlation between GBS epidemiology and the humoral immune response across various populations. The substantial variations in IgG concentration and functionality in different regions call for further exploration through a comprehensive system serology approach, including assessing antibody avidity, profiling glycosylation, and considering epigenetic factors. Finally, our study emphasizes the importance of expanding seroprevalence studies to include more diverse demographics for a comprehensive understanding of GBS immunity and for informing effective vaccine implementation strategies globally.

## Supplementary Data

Supplementary materials are available at *The Journal of Infectious Diseases* online (http://jid.oxfordjournals.org/). Supplementary materials consist of data provided by the author that are published to benefit the reader. The posted materials are not copyedited. The contents of all supplementary data are the sole responsibility of the authors. Questions or messages regarding errors should be addressed to the author.

## Supplementary Material

jiae607_Supplementary_Data

## Notes

***Acknowledgment.*** We are grateful to Prof C. J. Baker for providing standard GBS reference serums and to Pfizer and MinervaX for supplying GBS CPS and protein antigens. We thank Dr Todd Swarthout for facilitating sample collection in Malawi and the Child Health Research Foundation in Bangladesh. Additionally, we thank the United Kingdom Health Security Agency Seroepidemiology Unit, Manchester, United Kingdom, for providing samples for the UK cohort.

***Author contributions.*** S. K., A. M., K. L. D., A. K., and N. F. conceptualized the study and designed the experiments. Z. B. A. and S. K. S. collected patient data and samples in Bangladesh. S. K., S. S., S. Lim, T. H., S. Leung, and J. L. conducted the experiments. S. K., S. Lim, T. H., S. Leung, J. L., A. M., K. L. D., A. K., and N. F. analyzed the data. S. K. drafted the manuscript, and S. K., S. S., T. H., A. G., A. M., K. L. D., A. K., and N. F. edited and revised it. All authors reviewed and approved the final version of the manuscript.

***Financial support.*** This work was supported by the Immunising Pregnant Women and Infants Network, funded by the Global Challenges Research Fund Networks in Vaccines Research and Development, which was cofunded by the Medical Research Council and Biotechnology and Biological Sciences Research Council (grant MR/R005990/2). This UK-funded award is part of the EDCTP2 program supported by the European Union. Funding was awarded to S. K. as a postdoctoral fellowship. The Meningitis Research Foundation (project 0801.0) funded sample collection in Malawi.

## References

[1] Seale AC, Bianchi-Jassir F, Russell NJ, et al Estimates of the burden of group B streptococcal disease worldwide for pregnant women, stillbirths, and children. Clin Infect Dis 2017; 65:S200–19. 29117332 10.1093/cid/cix664 PMC5849940

[2] Edmond KM, Kortsalioudaki C, Scott S, et al Group B streptococcal disease in infants aged younger than 3 months: systematic review and meta-analysis. Lancet 2012; 379:547–56. 22226047 10.1016/S0140-6736(11)61651-6

[3] Kwatra G, Cunnington MC, Merrall E, et al Prevalence of maternal colonisation with group B *Streptococcus*: a systematic review and meta-analysis. Lancet Infect Dis 2016; 16:1076–84. 27236858 10.1016/S1473-3099(16)30055-X

[4] Russell NJ, Seale AC, O’Driscoll M, et al Maternal colonization with group B *Streptococcus* and serotype distribution worldwide: systematic review and meta-analyses. Clin Infect Dis 2017; 65:S100–11. 29117327 10.1093/cid/cix658 PMC5848259

[5] Gray KJ, Kafulafula G, Matemba M, Kamdolozi M, Membe G, French N. Group B *Streptococcus* and HIV infection in pregnant women, Malawi, 2008–2010. Emerg Infect Dis 2011; 17:1932–5. 22000375 10.3201/eid1710.102008 PMC3310663

[6] Carreras-Abad C, To K-N, Ramkhelawon L, et al Detection of group B *Streptococcus* colonisation in pregnant women: comparison of two different culture methods and study of antimicrobial resistance patterns. J Infect 2021; 82:186–230. 10.1016/j.jinf.2021.01.001 33428989

[7] Kwatra G, Izu A, Cutland C, et al Prevalence of group B *Streptococcus* colonisation in mother and newborn dyads in low-income and middle-income South Asian and African countries: a prospective, observational study. Lancet Microbe 2024; 5:100897. 39178870 10.1016/S2666-5247(24)00129-0 PMC11464403

[8] O'Sullivan CP, Lamagni T, Patel D, et al Group B streptococcal disease in UK and Irish infants younger than 90 days, 2014–15: a prospective surveillance study. Lancet Infect Dis 2019; 19:83–90. 30497953 10.1016/S1473-3099(18)30555-3

[9] Gray KJ, Bennett SL, French N, Phiri AJ, Graham SM. Invasive group B streptococcal infection in infants, Malawi. Emerg Infect Dis 2007; 13:223–9. 17479883 10.3201/eid1302.060680 PMC2725867

[10] Doare LK, Heath TP. An overview of global GBS epidemiology. Vaccine 2013; 31(suppl 4):D7–D12. 23973349 10.1016/j.vaccine.2013.01.009

[11] Madhi SA, Dangor Z. Prospects for preventing infant invasive GBS disease through maternal vaccination. Vaccine 2017; 35:4457–60. 28237500 10.1016/j.vaccine.2017.02.025

[12] Madhi SA, Anderson AS, Absalon J, et al Potential for maternally administered vaccine for infant group B *Streptococcus*. N Engl J Med 2023; 389:215–27. 37467497 10.1056/NEJMoa2116045

[13] Saukkoriipi A, Silmon de Monerri NC, Toropainen M, et al Association between anti-capsular IgG levels at birth and risk of invasive group B *Streptococcus* disease in Finnish newborns: a retrospective case-control study. Lancet Microbe 2024; 5:689–96. 38679040 10.1016/S2666-5247(24)00038-7

[14] Kwatra G, Adrian PV, Shiri T, Buchmann EJ, Cutland CL, Madhi SA. Natural acquired humoral immunity against serotype-specific group B *Streptococcus* rectovaginal colonization acquisition in pregnant women. Clin Microbiol Infect 2015; 21:568.e13–21. 10.1016/j.cmi.2015.01.030 25680313

[15] Baker CJ, Carey VJ, Rench MA, et al Maternal antibody at delivery protects neonates from early onset group B streptococcal disease. J Infect Dis 2014; 209:781–8. 24133184 10.1093/infdis/jit549 PMC3923540

[16] Lin FY, Weisman LE, Azimi PH, et al Level of maternal IgG anti-group B *Streptococcus* type III antibody correlated with protection of neonates against early-onset disease caused by this pathogen. J Infect Dis 2004; 190:928–34. 15295698 10.1086/422756

[17] Le Doare K, Kampmann B, Vekemans J, et al Serocorrelates of protection against infant group B *Streptococcus* disease. Lancet Infect Dis 2019; 19:e162–71. 30683467 10.1016/S1473-3099(18)30659-5

[18] Dangor Z, Kwatra G, Pawlowski A, et al Association of infant rib and alp1 surface protein N-terminal domain immunoglobulin G and invasive group B streptococcal disease in young infants. Vaccine 2023; 41:1679–83. 36754766 10.1016/j.vaccine.2023.01.071 PMC9996286

[19] Larsson C, Lindroth M, Nordin P, Stålhammar-Carlemalm M, Lindahl G, Krantz I. Association between low concentrations of antibodies to protein alpha and rib and invasive neonatal group B streptococcal infection. Arch Dis Child Fetal Neonatal Ed 2006; 91:F403–8. 17056838 10.1136/adc.2005.090472 PMC2672751

[20] Le Doare K, Faal A, Jaiteh M, et al Association between functional antibody against group B *Streptococcus* and maternal and infant colonization in a Gambian cohort. Vaccine 2017; 35:2970–8. 28449969 10.1016/j.vaccine.2017.04.013 PMC5432431

[21] Wolf AS, Mitsi E, Jones S, et al Quality of antibody responses by adults and young children to 13-valent pneumococcal conjugate vaccination and *Streptococcus pneumoniae* colonisation. Vaccine 2022; 40:7201–10. 36210249 10.1016/j.vaccine.2022.09.069 PMC10615833

[22] Song JY, Moseley MA, Burton RL, Nahm MH. Pneumococcal vaccine and opsonic pneumococcal antibody. J Infect Chemother 2013; 19:412–25. 23657429 10.1007/s10156-013-0601-1 PMC3692352

[23] Pawlowski A, Lannergård J, Gonzalez-Miro M, et al A group B *Streptococcus* alpha-like protein subunit vaccine induces functionally active antibodies in humans targeting homotypic and heterotypic strains. Cell Rep Med 2022; 3:100511. 35243418 10.1016/j.xcrm.2022.100511 PMC8861819

[24] Vidarsson G, Dekkers G, Rispens T. IgG subclasses and allotypes: from structure to effector functions. Front Immunol 2014; 5:520. 25368619 10.3389/fimmu.2014.00520 PMC4202688

[25] Firan M, Bawdon R, Radu C, et al The MHC class I-related receptor, FcRn, plays an essential role in the maternofetal transfer of gamma-globulin in humans. Int Immunol 2001; 13:993–1002. 11470769 10.1093/intimm/13.8.993

[26] Baker JA, Lewis EL, Byland LM, Bonakdar M, Randis TM, Ratner AJ. Mucosal vaccination promotes clearance of *Streptococcus agalactiae* vaginal colonization. Vaccine 2017; 35:1273–80. 28162823 10.1016/j.vaccine.2017.01.029 PMC5319564

[27] Buurman ET, Timofeyeva Y, Gu J, et al A novel hexavalent capsular polysaccharide conjugate vaccine (GBS6) for the prevention of neonatal group B streptococcal infections by maternal immunization. J Infect Dis 2019; 220:105–15. 30778554 10.1093/infdis/jiz062 PMC6548902

[28] Absalon J, Segall N, Block SL, et al Safety and immunogenicity of a novel hexavalent group B *Streptococcus* conjugate vaccine in healthy, non-pregnant adults: a phase 1/2, randomised, placebo-controlled, observer-blinded, dose-escalation trial. Lancet Infect Dis 2021; 21:263–74. 32891191 10.1016/S1473-3099(20)30478-3 PMC9760110

[29] Gaylord MA, Larrier M, Giordano-Schmidt D, et al Development and validation of a 6-plex Luminex-based assay for measuring human serum antibodies to group B *Streptococcus* capsular polysaccharides. Hum Vaccin Immunother 2024; 20:2311480. 38608171 10.1080/21645515.2024.2311480 PMC11018021

[30] Fischer P, Pawlowski A, Cao D, et al Safety and immunogenicity of a prototype recombinant alpha-like protein subunit vaccine (GBS-NN) against group B *Streptococcus* in a randomised placebo-controlled double-blind phase 1 trial in healthy adult women. Vaccine 2021; 39:4489–99. 34215454 10.1016/j.vaccine.2021.06.046

[31] Leung S, Collett CF, Allen L, et al Development of A standardized opsonophagocytosis killing assay for group B *Streptococcus* and assessment in an interlaboratory study. Vaccines (Basel) 2023; 11:1703. 38006035 10.3390/vaccines11111703 PMC10675794

[32] Saha SK, Ahmed ZB, Modak JK, et al Group B *Streptococcus* among pregnant women and newborns in Mirzapur, Bangladesh: colonization, vertical transmission, and serotype distribution. J Clin Microbiol 2017; 55:2406–12. 28515218 10.1128/JCM.00380-17 PMC5527418

[33] Bianchi-Jassir F, Paul P, To KN, et al Systematic review of group B streptococcal capsular types, sequence types and surface proteins as potential vaccine candidates. Vaccine 2020; 38:6682–94. 32888741 10.1016/j.vaccine.2020.08.052 PMC7526974

[34] Hall J, Adams NH, Bartlett L, et al Maternal disease with group B *Streptococcus* and serotype distribution worldwide: systematic review and meta-analyses. Clin Infect Dis 2017; 65:S112–24. 29117328 10.1093/cid/cix660 PMC5850000

[35] Islam MS, Saha SK, Islam M, et al Prevalence, serotype distribution and mortality risk associated with group B *Streptococcus* colonization of newborns in rural Bangladesh. Pediatr Infect Dis J 2016; 35:1309–12. 27455441 10.1097/INF.0000000000001306

[36] Chaguza C, Jamrozy D, Bijlsma MW, et al Population genomics of group B *Streptococcus* reveals the genetics of neonatal disease onset and meningeal invasion. Nat Commun 2022; 13:4215. 35864107 10.1038/s41467-022-31858-4 PMC9304382

[37] Fabbrini M, Rigat F, Rinaudo CD, et al The protective value of maternal group B *Streptococcus* antibodies: quantitative and functional analysis of naturally acquired responses to capsular polysaccharides and pilus proteins in European maternal sera. Clin Infect Dis 2016; 63:746–53. 27402816 10.1093/cid/ciw377

[38] Haeusler IL, Daniel O, Isitt C, et al Group B *Streptococcus* (GBS) colonization is dynamic over time, whilst GBS capsular polysaccharides-specific antibody remains stable. Clin Exp Immunol 2022; 209:188–200. 35802786 10.1093/cei/uxac066 PMC9390841

[39] McGee L, Chochua S, Li Z, et al Multistate, population-based distributions of candidate vaccine targets, clonal complexes, and resistance features of invasive group B streptococci within the United States, 2015–2017. Clin Infect Dis 2021; 72:1004–13. 32060499 10.1093/cid/ciaa151 PMC8071603

[40] Maeland JA, Afset JE, Lyng RV, Radtke A. Survey of immunological features of the alpha-like proteins of *Streptococcus agalactiae*. Clin Vaccine Immunol 2015; 22:153–9. 25540270 10.1128/CVI.00643-14 PMC4308872

[41] Givner LB, Baker CJ, Edwards MS. Type III group B *Streptococcus*: functional interaction with IgG subclass antibodies. J Infect Dis 1987; 155:532–9. 3543156 10.1093/infdis/155.3.532

[42] Saeland E, Vidarsson G, Leusen JH, et al Central role of complement in passive protection by human IgG1 and IgG2 anti-pneumococcal antibodies in mice. J Immunol 2003; 170:6158–64. 12794146 10.4049/jimmunol.170.12.6158

[43] Simell B, Nurkka A, Ekström N, Givon-Lavi N, Käyhty H, Dagan R. Serum IgM antibodies contribute to high levels of opsonophagocytic activities in toddlers immunized with a single dose of the 9-valent pneumococcal conjugate vaccine. Clin Vaccine Immunol 2012; 19:1618–23. 22875604 10.1128/CVI.00248-12 PMC3485875

[44] Muri L, Schubart A, Thorburn C, et al Inhibition of the different complement pathways has varying impacts on the serum bactericidal activity and opsonophagocytosis against *Haemophilus influenzae* type b. Front Immunol 2022; 13:1020580. 36578495 10.3389/fimmu.2022.1020580 PMC9791579

[45] Chen X, Shi M, Tong X, et al Glycosylation-dependent opsonophagocytic activity of staphylococcal protein A antibodies. Proc Natl Acad Sci U S A 2020; 117:22992–3000. 32855300 10.1073/pnas.2003621117 PMC7502815

[46] Anttila M, Voutilainen M, Jäntti V, Eskola J, Käyhty H. Contribution of serotype-specific IgG concentration, IgG subclasses and relative antibody avidity to opsonophagocytic activity against *Streptococcus pneumoniae*. Clin Exp Immunol 1999; 118:402–7. 10594558 10.1046/j.1365-2249.1999.01077.x PMC1905437

[47] Rio-Aige K, Azagra-Boronat I, Castell M, et al The breast milk immunoglobulinome. Nutrients 2021; 13:1810. 34073540 10.3390/nu13061810 PMC8230140

[48] Dangor Z, Kwatra G, Izu A, et al HIV-1 is associated with lower group B *Streptococcus* capsular and surface-protein IgG antibody levels and reduced transplacental antibody transfer in pregnant women. J Infect Dis 2015; 212:453–62. 25651843 10.1093/infdis/jiv064

[49] Office for National Statistics . How life has changed in Exeter: census. 2021. Available at: https://www.ons.gov.uk/visualisations/censusareachanges/E07000041/. Accessed 29 February 2024.

