# Supplementary material for: Diversity in Naturally Acquired Immunity to Group B Streptococcus: A Comparative Study of Women From Bangladesh, Malawi, and the United Kingdom
Source: J Infect Dis. 2024 Dec 18;231(2):e456–67. doi: 10.1093/infdis/jiae607 (PMC11841642; doi:10.1093/infdis/jiae607)
Supplement: jiae607_Supplementary_Data [file jiae607_supplementary_data.pdf]

Supplementary Materials

Khandaker et al.: Diversity in naturally acquired immunity to Group B Streptococcus: A Comparative Study of Women from Bangladesh, Malawi, and the United Kingdom

Supplementary Methods

Ethics for the clinical study.

Serum samples from Malawi were sourced from the NET-GBS study (Reference no: P.05/14/1574), and the STREPCAR study (ethics no: P.07/08/686) conducted in Blantyre, Malawi, from 2008 to 2016. Bangladeshi samples were obtained from women attending the Kumudini Women's Medical College Hospital in Mirzapur, Bangladesh, from 2019 to 2020 (Study reference no: BICH-ERC-03-05-2018). Serum samples from the UK were derived from the United Kingdom Health Security Agency (UKHSA) serum collection, containing the residues of specimens submitted for diagnostic testing from 2015 to 2018. Ethical approval was granted by local committees from each study area and the University of Liverpool Health and Life Sciences Research Ethics Committee (Ethics reference number: 4797).

Quantitation of CPS-specific IgG in sera.

MagPlex microspheres (Luminex Corp) were coated with CPS-poly-l-lysine conjugates, GBS serotypes Ia, Ib, II, III, IV and V (Pfizer). An 11-point standard curve, diluted 1/50 and serially diluted 2.5-fold, QC samples and blank wells were included on each plate. All samples and controls were diluted into assay buffer (10mMPBS/0.5% BSA/0.05% Tween/0.02% Sodium Azide, pH 7.2) and combined with GBS CPS-PLL-coupled microspheres (5×10^4 microspheres/ml per serotype, 50 µL). Samples and QC samples were diluted to 1/500, 1/5000, and 1/50000.

Assay plates were mixed on an orbital shaker (MaxQ 2000 shaker, 300 RPM) and incubated overnight at 4°C. The following day, plates were washed with wash buffer (1xPBS/0.05% Tween-20/0.02% Sodium Azide, pH 7.2), followed by a 1.5-hour incubation with a 1/500 dilution of R-Phycoerythrin-conjugated goat α-human IgG Fcγ-specific antibody (109-115-098, Jackson ImmunoResearch, UK) at

room temperature with agitation. After the final wash and resuspension in 100 µL of wash buffer, antigen-IgG binding was measured using a Bioplex-200 (Bio-Rad). The results were obtained as median fluorescence intensities (MFIs), later converted to concentrations (µg/ml) based on the standard curve interpolation. IgG assay lower limit of quantifications (LLOQ) was assigned according to Gaylord et al., 2024. (1)

Quantitation of Alp-N-specific IgG in sera. Serum IgG Abs to GBS surface protein antigens (Alp1-N, Alp2/3-N, αC-N, and Rib-N) were quantified using ELISA. A MinervaX internal reference serum was utilised as a standard with the known concentration of antibodies specific to each Alp-N protein. ELISA plates were coated with 0.5 µg/ml of relevant recombinant Alp protein (Bioneer A/S) by incubating overnight at 4°C. The following day, the plates were washed and blocked with PBS-3 % BSA (blocking buffer) for 1 hour at room temperature. Serum samples and MinervaX reference serum were serially diluted in PBS-3 % BSA-0.05% Tween 20 (sample buffer) and added to the plates. Following a 2-hour incubation at room temperature, horseradish peroxidase (HRP)-conjugated detection antibody, Goat F(ab')2 Anti-Human IgG-HRP (SouthernBiotech 2042-05), was added and incubated for an additional hour at room temperature. HRP was detected using 100 µl/well of 3, 3', 5, 5'-tetramethylbenzidine substrate (TMB PLUS2 ELISA HRP Substrate, Kementec 4395) and the resulting colour reaction was stopped with 1 M sulfuric acid after a 30 min incubation at room temperature. Absorbance readings were taken at 450 nm wavelength using a Spectrostar Nano microplate reader (BMG Labtech). Quantification of antibody concentrations in serum samples was performed for all sample dilutions by referencing the absorbance values obtained from the calibrated MinervaX reference serum standard curves. IgG assay LLOQs (µg/ml) for each Alp protein in this study were: Alp1-N = 0.0076, Alp2/3-N = 0.01, αC-N = 0.0078, and Rib-N = 0.0042.

Opsonophagocytosis killing assay (OPKA). HL-60 cells (CCL-240; American Type Culture Collection) were cultured in RPMI medium (Sigma Aldrich, Burlington, MA, USA) containing 2 mM L-glutamine and 20% heat-inactivated foetal bovine serum (LabTech, Heathfield, UK). These cells were differentiated into neutrophil-like cells over five days, maintaining a cell density of 5×10^5 cells/ml in

RPMI medium supplemented with 0.8% dimethylformamide (Sigma Aldrich, Burlington, MA, USA). The differentiation of HL-60 cells was confirmed using flow cytometry with fluorescently labelled mouse/rat anti-human monoclonal antibodies targeting cell surface markers CD11b, CD55, and CD71 (BioLegend). Differentiated cells meeting the criteria (>55% CD35+ and CD11b+ and <20% CD71+) were used in the OPK assay.

In the OPK assay, serum samples were serially diluted in a 96-well plate and then incubated with 1×10^4/well GBS serotype Ib (NCTC, 14092) for 30 minutes at 37°C. Subsequently, differentiated HL-60 cells (1×10^6/well), pre-washed in Hank's balanced salt solution (HBSS), were combined with 12.5% baby rabbit complement and added to the serum-bacteria mixture. Following another 30-minute incubation at 37°C, a 10μl assay mixture was plated onto COH agar, forming streaks by tilting. The plates were then incubated overnight at 37°C with 5% CO2, and the bacterial colonies were counted manually or using digital image analysis methods. The killing titres were determined to be at the 50% reciprocal dilution point compared to the average Colony-Forming Units (CFU) observed in serum-free controls.

Supplementary Results

Table S1: Details of selected serum samples from three countries to construct the serum pool for passive immunisation in mice.

<table>
<tr><th></th><th>Sample ID</th><th>CPS Ib IgG (µg/ml)</th><th>Volume taken</th><th>Final concentration (normalised with PBS)</th></tr>
<tr><td rowspan="3">UK</td><td>M219061</td><td>10.925</td><td>400µl</td><td rowspan="3">6 µg/ml</td></tr>
<tr><td>M190615</td><td>5.340</td><td>400µl</td></tr>
<tr><td>M202044</td><td>1.771</td><td>400µl</td></tr>
<tr><td rowspan="3">Bangladesh</td><td>85138</td><td>9.798</td><td>400µl</td><td rowspan="3">6 µg/ml</td></tr>
<tr><td>87948</td><td>2.643</td><td>400µl</td></tr>
<tr><td>85502</td><td>5.583</td><td>400µl</td></tr>
<tr><td rowspan="2">Malawi</td><td>672</td><td>6.727</td><td>1000µl</td><td rowspan="2">6 µg/ml</td></tr>
<tr><td>802</td><td>2.493</td><td>200µl</td></tr>
</table>

Table S2: Geometric Mean Concentration (GMC) of IgG to CPS Ia, Ib, II, III, IV, and V of Group B Streptococcus in the UK, Bangladesh, and Malawi along with the inter-country comparisons of the IgG concentrations.

Country	Geometric mean concentration (GMC)	95% CI	Comparator countries	Adjusted P Values*
	**CPS Ia**			
UK	0.034	0.017-0.065	UK vs. BD	<0.0001
BD	0.929	0.533-1.617	UK vs. Malawi	0.0970
Malawi	0.011	0.006-0.018	BD vs. Malawi	<0.0001
	**CPS Ib**			
UK	0.015	0.009-0.023	UK vs. BD	<0.0001
BD	0.099	0.063-0.154	UK vs. Malawi	>0.9999
Malawi	0.017	0.011-0.026	BD vs. Malawi	<0.0001
	**CPS II**			
UK	0.268	0.171-0.420	UK vs. BD	<0.0001
BD	2.306	1.613-3.298	UK vs. Malawi	0.0905
Malawi	0.579	0.405-0.827	BD vs. Malawi	<0.0001
	**CPS III**			
UK	0.047	0.029-0.076	UK vs. BD	<0.0001
BD	0.472	0.299-0.745	UK vs. Malawi	0.2558
Malawi	0.024	0.016-0.037	BD vs. Malawi	<0.0001
	**CPS IV**			
UK	0.014	0.010-0.021	UK vs. BD	0.0011
BD	0.034	0.023-0.051	UK vs. Malawi	0.1398
Malawi	0.021	0.015-0.030	BD vs. Malawi	0.4300

	**CPS V**			
UK	0.012	0.008-0.018	UK vs. BD	0.0003
BD	0.032	0.020-0.050	UK vs. Malawi	0.1459
Malawi	0.014	0.011-0.020	BD vs. Malawi	0.2352

Table S3: Geometric Mean Concentration (GMC) of IgG directed against Alp1-N, Alp2/3-N, aC-N and Rib-N proteins in the UK, Bangladesh, and Malawi along with the inter-country comparisons of the IgG concentrations.

Country	Geometric mean concentration (GMC)	95% CI	Comparator countries	Adjusted P Value*
	**Alp1-N**			
UK	0.087	0.071-0.108	UK vs. BD	>0.9999
BD	0.082	0.067-0.099	UK vs. Malawi	0.0156
Malawi	0.057	0.046-0.068	BD vs. Malawi	0.0391
	**Alp2/3-N**			
UK	0.090	0.070-0.116	UK vs. BD	>0.9999
BD	0.079	0.065-0.097	UK vs. Malawi	<0.0001
Malawi	0.038	0.032-0.045	BD vs. Malawi	<0.0001
	**aC-N**			
UK	0.070	0.055-0.089	UK vs. BD	>0.9999
BD	0.064	0.053-0.079	UK vs. Malawi	0.0294
Malawi	0.044	0.036-0.052	BD vs. Malawi	0.0346
	**Rib-N**			
UK	0.061	0.051-0.074	UK vs. BD	0.4024
BD	0.073	0.061-0.086	UK vs. Malawi	0.3375
Malawi	0.049	0.041-0.058	BD vs. Malawi	0.0062

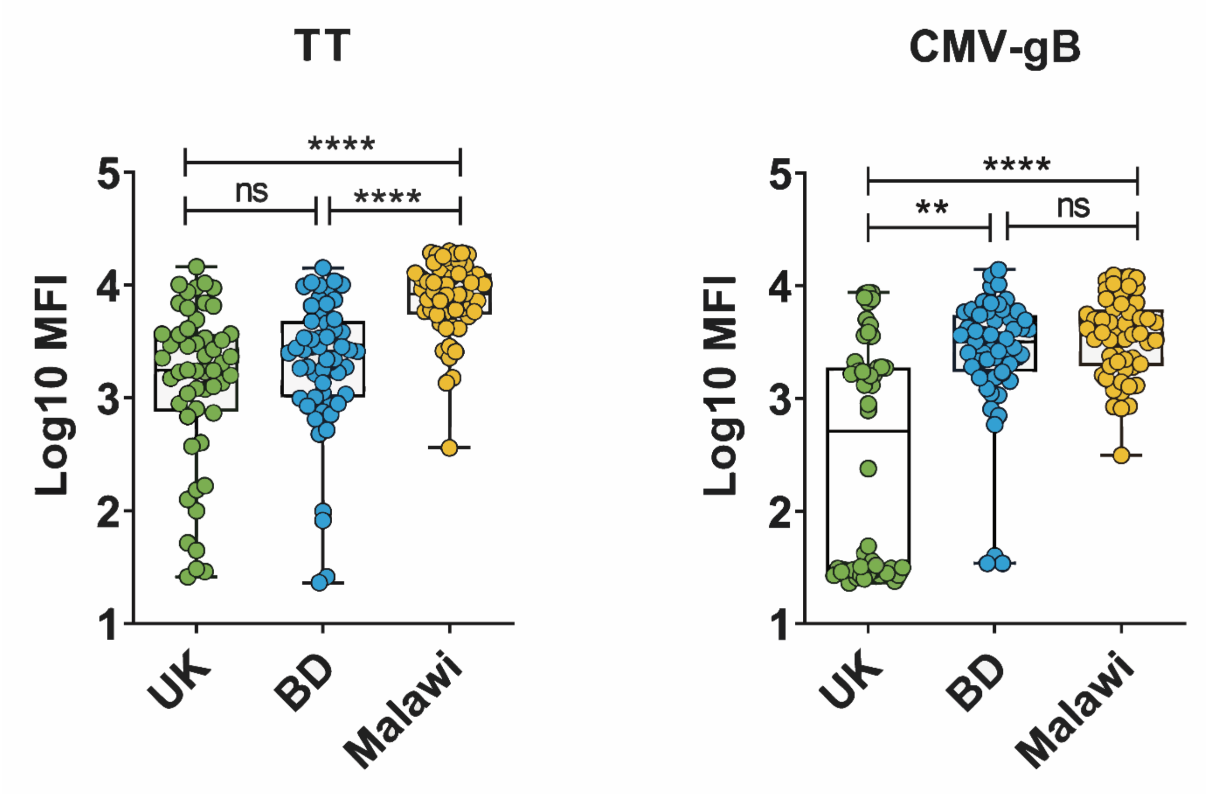

Figure S1. Serum concentration of IgG against Tetanus Toxoid (TT) and Cytomegalovirus Glycoprotein B (CMV-gB) antigens in women in the UK, Bangladesh, and Malawi.

The IgG concentration against TT and CMV-gB was determined using the multiplexed Luminex bead-based method. Concentrations are expressed in Mean Fluorescence Intensity (MFI). Statistical significance was determined using the Kruskal-Wallis and Dann's multiple comparison tests. (**p<0.01, ****p<0.0001, 'ns' used for non-significant differences). Box and Whisker plots indicate median, IQR and minimum/maximum values.

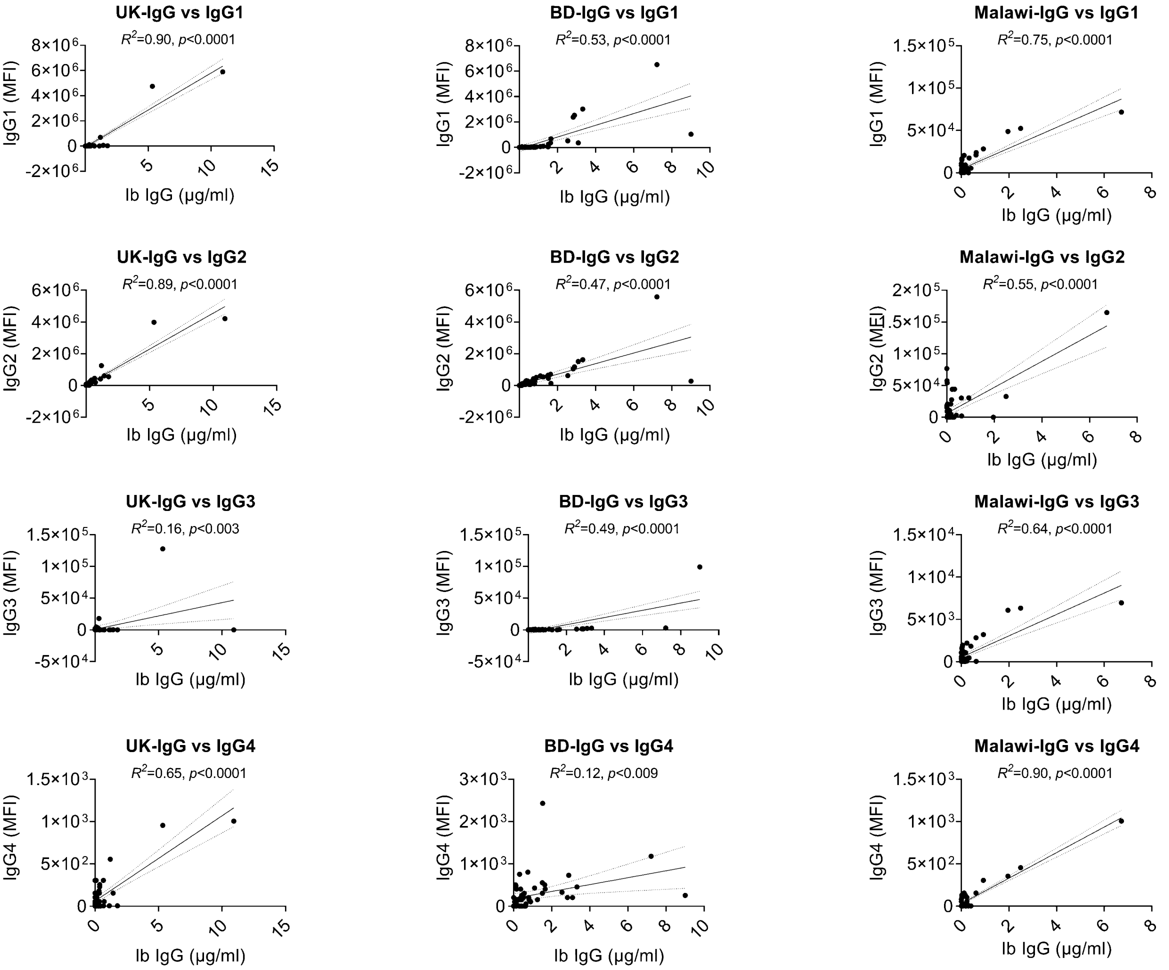

Figure S2. Correlation between anti-CPS Ib IgG titre and the subclasses. Simple linear regression was performed to assess the relationship between CPS Ib-specific IgG titres (µg/ml) and subclasses (IgG1, IgG2, IgG3, and IgG4) expressed in mean fluorescent intensity (MFI). The figure illustrates the correlations, including 95% confidence bands of the best fit.

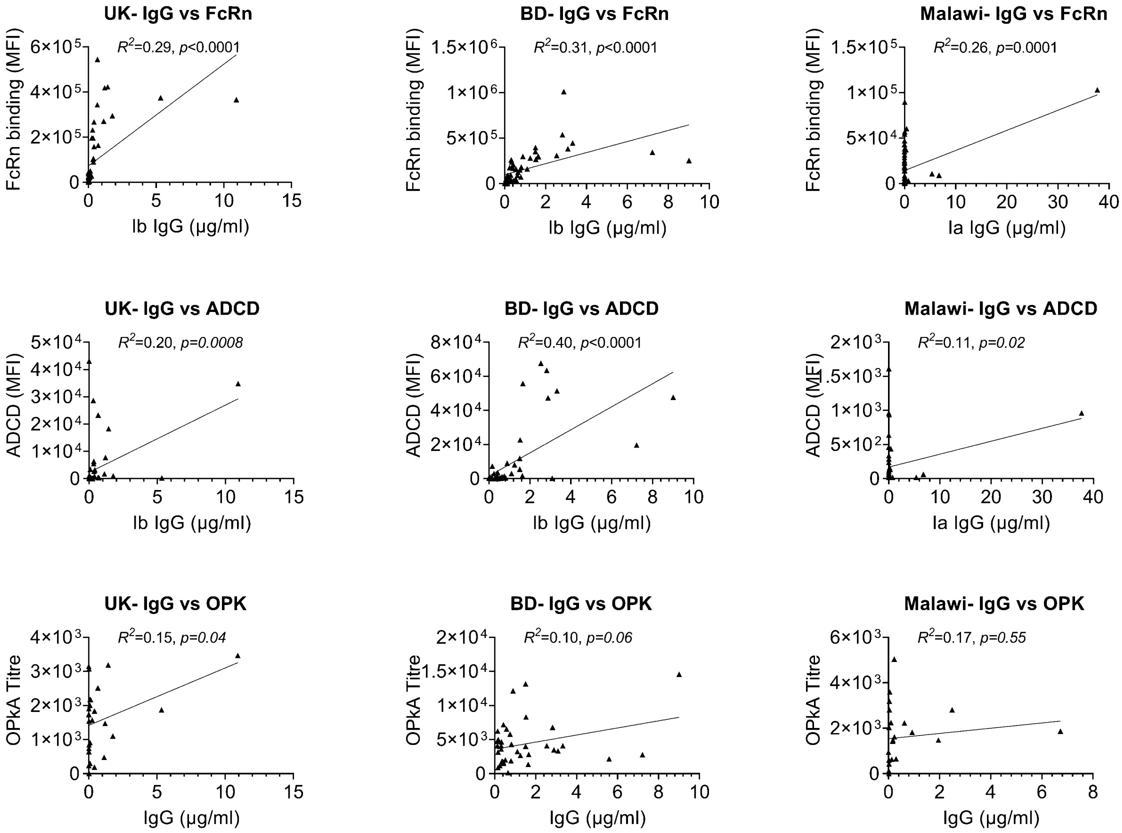

Figure S3. Correlation between anti-CPS Ib IgG titre and functional properties. Simple linear regression was performed to assess the relationship between CPS Ib-specific IgG titres (µg/ml) and FcRn receptor binding, antibody-dependent complement deposition (ADCD), and opsonophagocytic killing (OPK). The figure illustrates the correlations, including 95% confidence bands of the best fit.

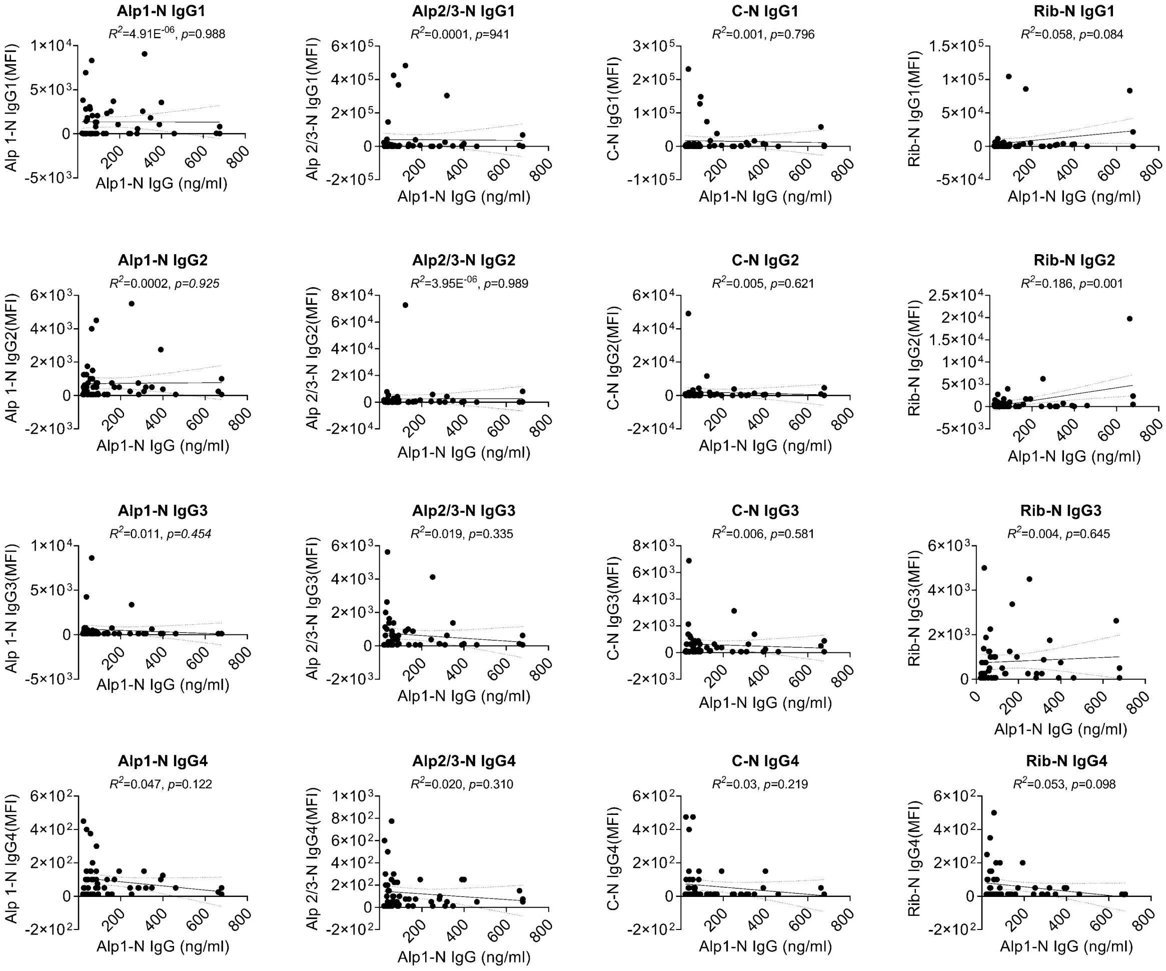

Figure S4. Correlation Between Alp Protein-Specific IgG Titres and Subclasses in Sera from the UK. Simple linear regression was performed to assess the relationship between IgG titres (ng/ml) and subclasses (IgG1, IgG2, IgG3, and IgG4) expressed in mean fluorescent intensity (MFI). The figure illustrates the correlations, including 95% confidence bands of the best fit.

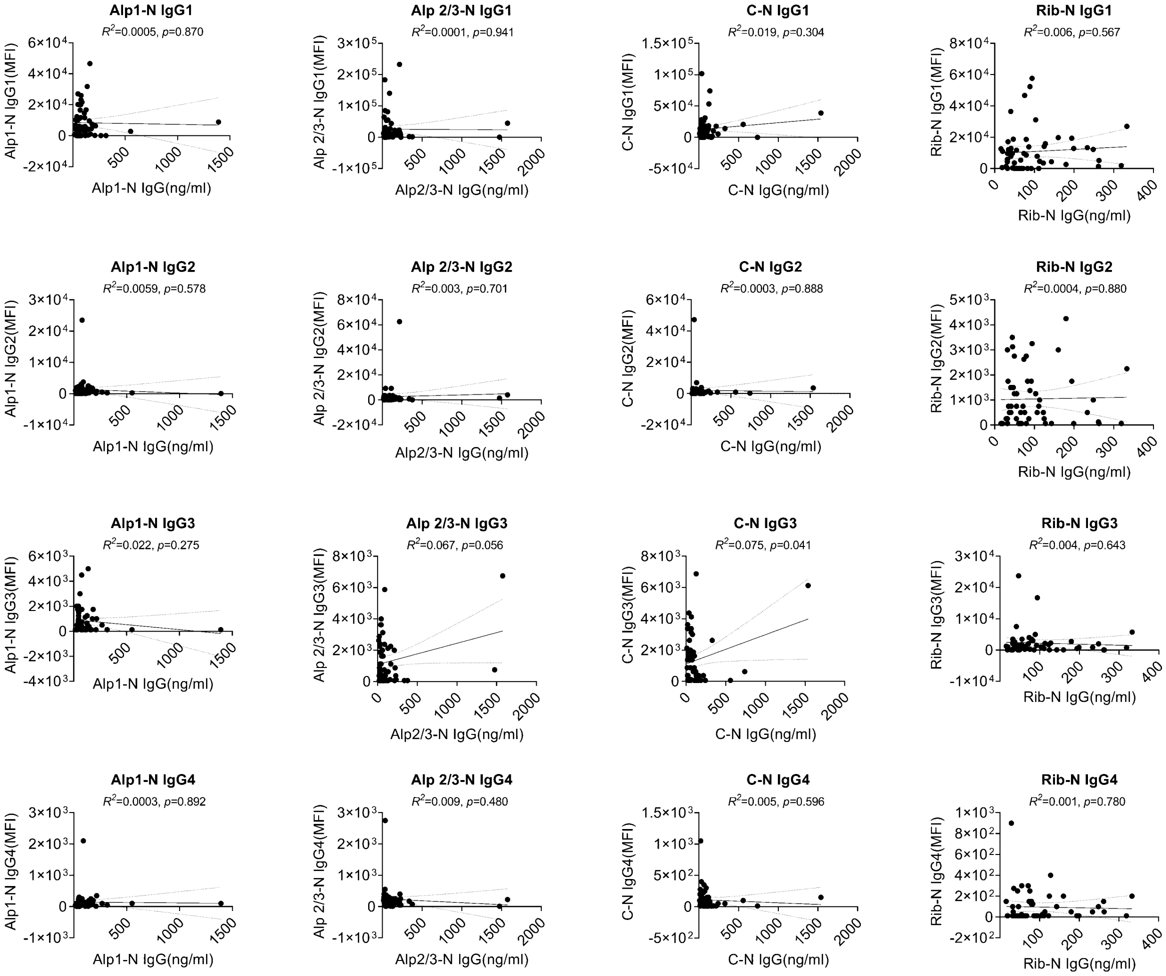

Figure S5. Correlation Between Alp Protein-Specific IgG Titres and Subclasses in Sera from Bangladesh. Simple linear regression was performed to assess the relationship between IgG titres (ng/ml) and subclasses (IgG1, IgG2, IgG3, and IgG4) expressed in mean fluorescent intensity (MFI). The figure illustrates the correlations, including 95% confidence bands of the best fit.

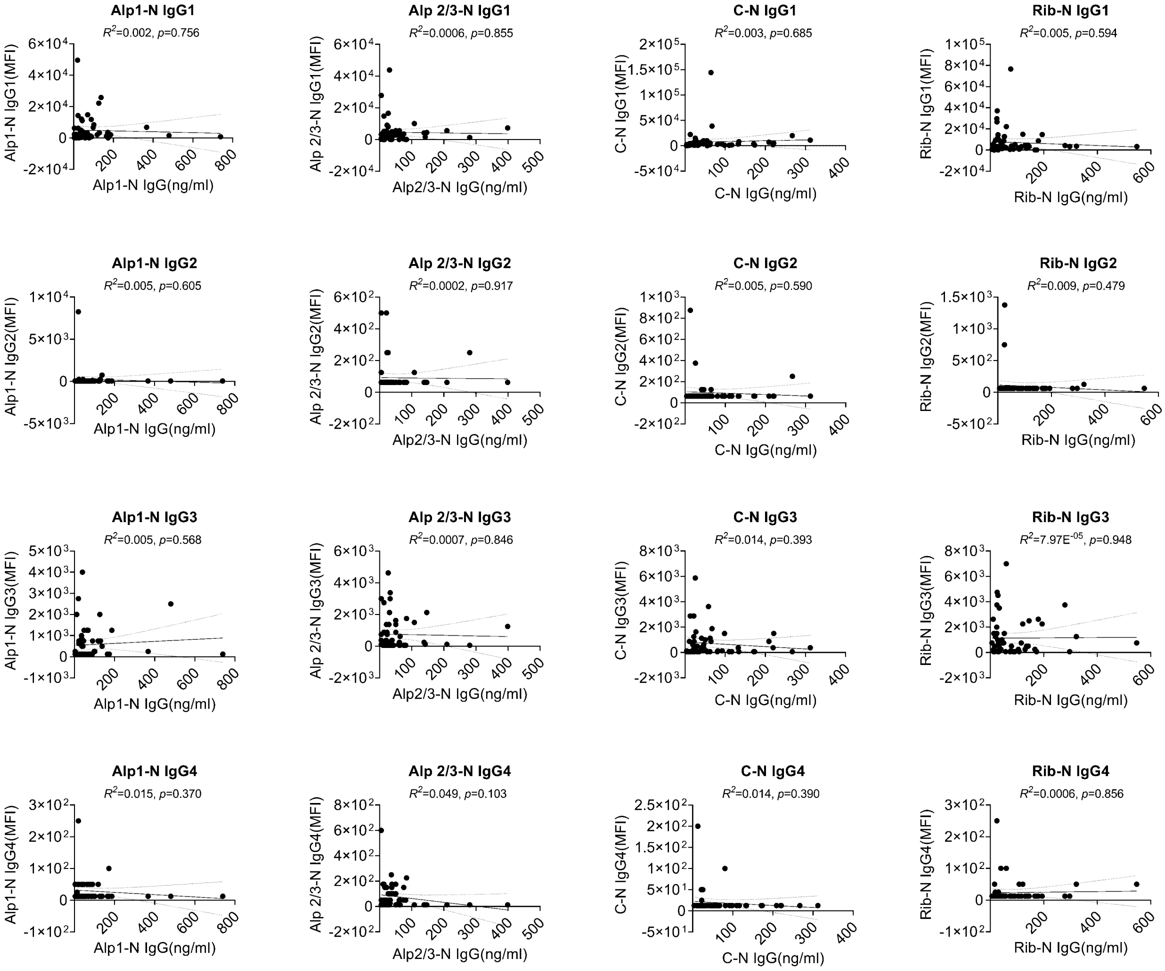

Figure S6. Correlation Between Alp Protein-Specific IgG Titres and Subclasses in Sera from Malawi. Simple linear regression was performed to assess the relationship between IgG titres (ng/ml) and subclasses (IgG1, IgG2, IgG3, and IgG4) expressed in mean fluorescent intensity (MFI). The figure illustrates the correlations, including 95% confidence bands of the best fit.

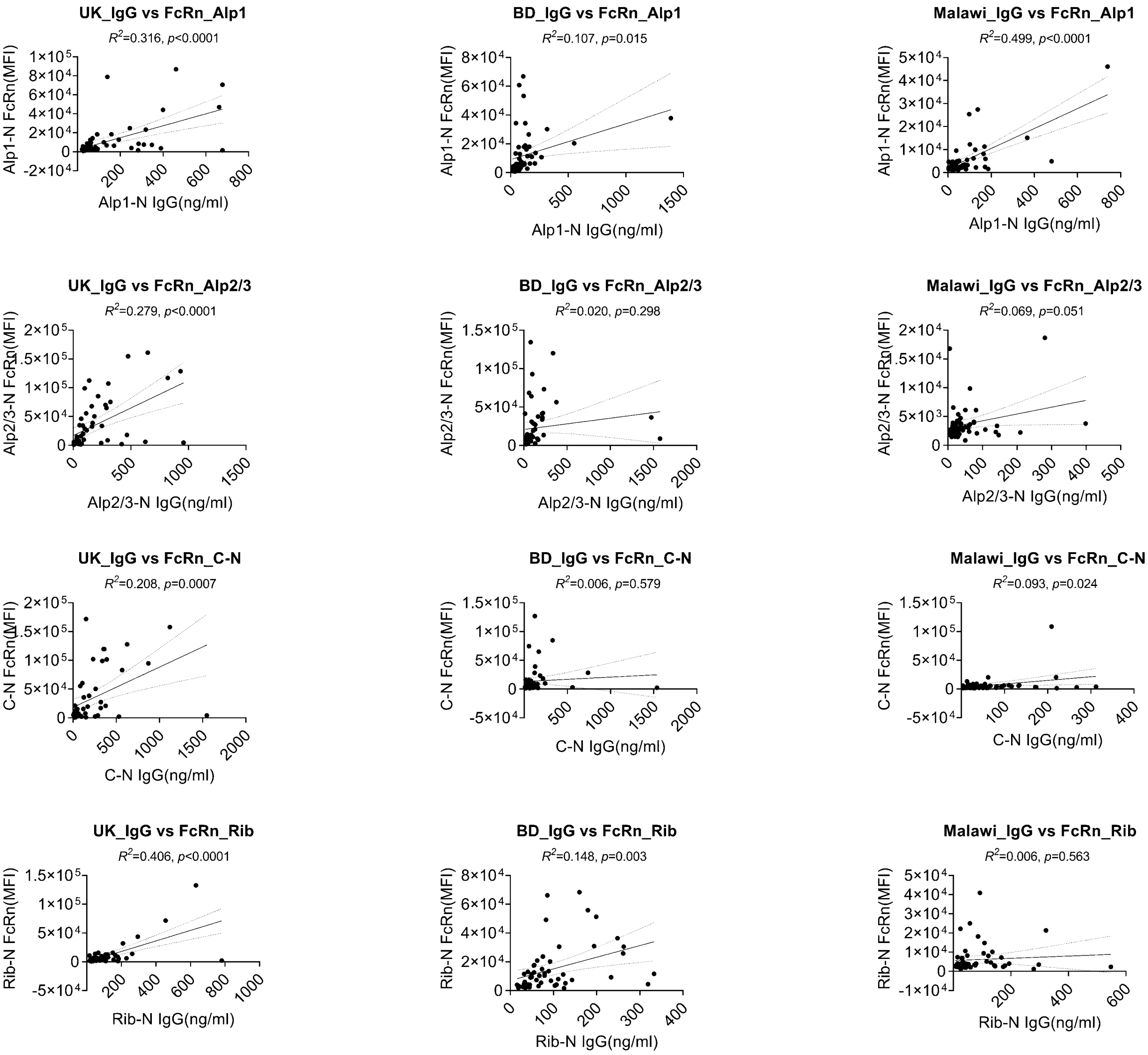

Figure S7. Correlation between Alp Protein-Specific IgG titre and FcRn binding. Simple linear regression was performed to assess the relationship between four Alp protein-Specific IgG titre (ng/ml) and FcRn binding expressed in mean fluorescent intensity (MFI). The figure illustrates the correlations, including 95% confidence bands of the best fit.

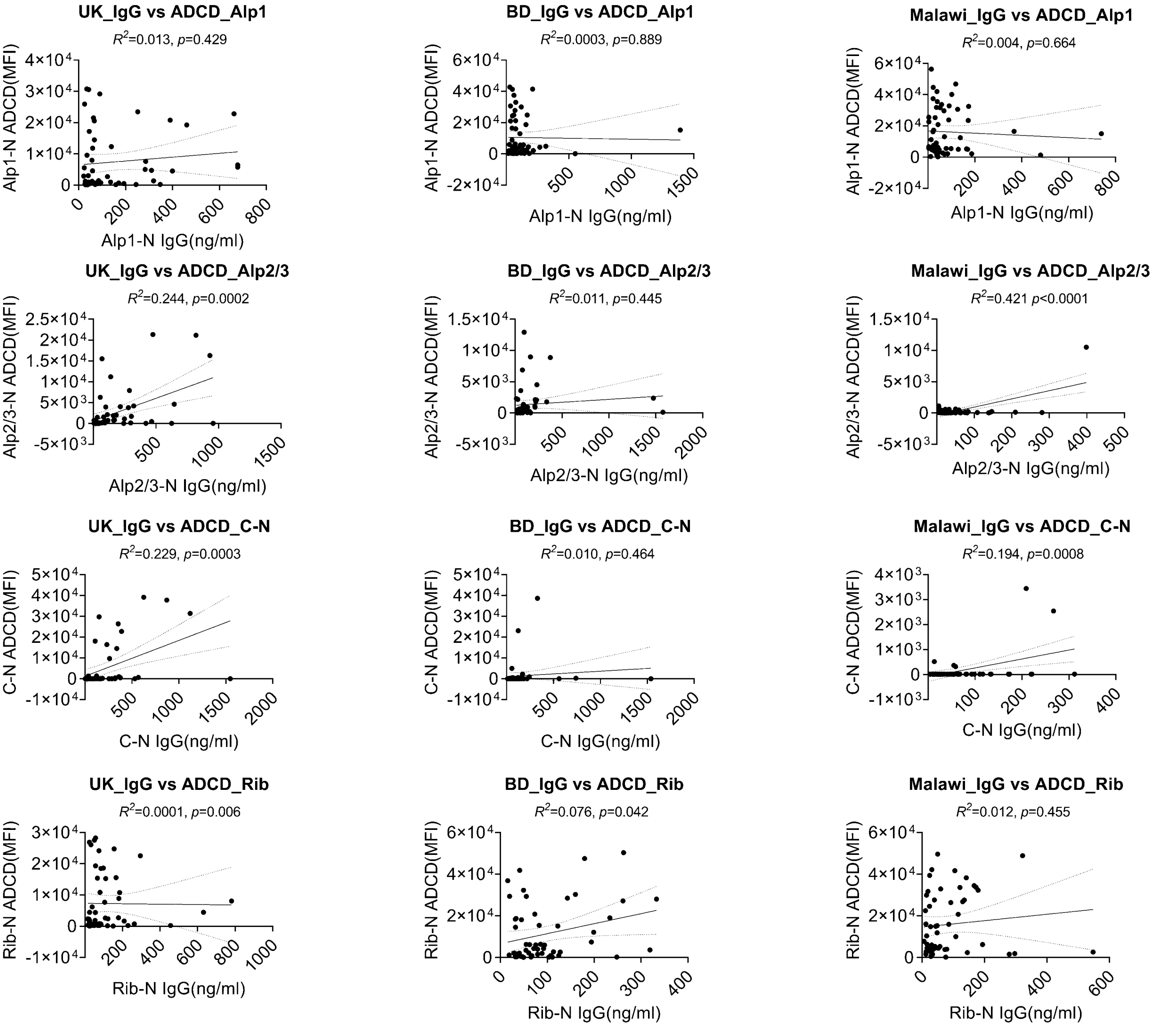

Figure S8. Correlation between Alp Protein-Specific IgG titre and antibody-dependent complement deposition (ADCD). Simple linear regression was performed to assess the relationship between four Alp protein-Specific IgG titre (ng/ml) and antibody-dependent complement deposition (ADCD) expressed in mean fluorescent intensity (MFI). The figure illustrates the correlations, including 95% confidence bands of the best fit.

References:

1. Gaylord MA, Larrier M, Giordano-Schmidt D, Grube CD, Singh S, Nguyen HH, et al. Development and validation of a 6-plex Luminex-based assay for measuring human serum antibodies to group B streptococcus capsular polysaccharides. Hum Vaccin Immunother. 2024;20(1):2311480.
